# Supplementary material for: Retrieval practice facilitates memory updating by enhancing and differentiating medial prefrontal cortex representations
Source: eLife. 2020 May 18;9:e57023. doi: 10.7554/eLife.57023 (PMC7272192; doi:10.7554/eLife.57023)
Supplement: Supplementary file 1. [file elife-57023-supp1.docx]

Supplementary File 1. Update method (RetPrac, Restudy) X Test order (A-C first, A-B first) 2-way ANOVA table by Memory test type and Response type

| Memory test type | Response Type | Effect | F | df | P (raw) | P |
| --- | --- | --- | --- | --- | --- | --- |
| Recall A-C | Target | Update method | 15.19 | 1,44 | <.001 | .002 |
|  |  | Test order | 0.16 | 1,44 | .689 | .827 |
|  |  | Update method X Test order | 0.13 | 1,44 | .718 | .805 |
|  | Competitor | Update method | 7.57 | 1,44 | .009 | .027 |
|  |  | Test order | 0.54 | 1,44 | .466 | .827 |
|  |  | Update method X Test order | 0.97 | 1,44 | .329 | .658 |
|  | Other | Update method | 2.96 | 1,44 | .093 | .140 |
|  |  | Test order | 0.17 | 1,44 | .679 | .827 |
|  |  | Update method X Test order | 0.37 | 1,44 | .549 | .805 |
| Recall A-B | Target | Update method | 0.63 | 1,44 | .432 | .432 |
|  |  | Test order | 0.03 | 1,44 | .874 | .874 |
|  |  | Update method X Test order | 0.06 | 1,44 | .805 | .805 |
|  | Competitor | Update method | 1.86 | 1,44 | .180 | .216 |
|  |  | Test order | 2.21 | 1,44 | .144 | .432 |
|  |  | Update method X Test order | 2.47 | 1,44 | .123 | .540 |
|  | Other | Update method | 5.19 | 1,44 | .028 | .056 |
|  |  | Test order | 7.00 | 1,44 | .011 | .066 |
|  |  | Update method X Test order | 1.85 | 1,44 | .180 | .540 |

Note: P values were FDR adjusted for multiple comparisons.
