# Supplementary material for: Retrieval practice facilitates memory updating by enhancing and differentiating medial prefrontal cortex representations
Source: eLife. 2020 May 18;9:e57023. doi: 10.7554/eLife.57023 (PMC7272192; doi:10.7554/eLife.57023)
Supplement: Supplementary file 2. — (a) Updating method (RetPrac, Restudy) x Classifier evidence (Target, Competitor) x Memory outcome (Correct, Incorrect) three-way ANOVA table. (b) Updating method (RetPrac, Restudy) x Memory outcome (Correct, Incorrect) two-way ANOVA table by Classifier evidence type. [file elife-57023-supp2.docx]

Supplementary File 2a. Updating method (RetPrac, Restudy) X Classifier evidence (Target, Competitor) X Memory outcome (Correct, Incorrect) 3-way ANOVA table.

| ROI | Effect | F | df | P |
| --- | --- | --- | --- | --- |
| MPFC | Update method | 2.07 | 1,18 | .17 |
|  | Classifier evidence | 0.26 | 1,18 | .62 |
|  | Memory outcome | 0.51 | 1,18 | .48 |
|  | Update method X Classifier evidence | 6.50 | 1,18 | .02 |
|  | Update method X memory outcome | 0.77 | 1,18 | .39 |
|  | Classifier evidence X memory outcome | 23.25 | 1,18 | < .001 |
|  | Update method X Classifier evidence X Memory outcome | 3.34 | 1,18 | .08 |
| VTC | Update method | 5.58 | 1,18 | .03 |
|  | Classifier evidence | 5.03 | 1,18 | .04 |
|  | Memory outcome | 6.95 | 1,18 | .02 |
|  | Update method X Classifier evidence | 0.16 | 1,18 | .70 |
|  | Update method X memory outcome | 1.79 | 1,18 | .20 |
|  | Classifier evidence X memory outcome | 26.51 | 1,18 | < .001 |
|  | Update method X Classifier evidence X Memory outcome | 0.22 | 1,18 | .65 |
| AG | Update method | 0.01 | 1,18 | .99 |
|  | Classifier evidence | 0.11 | 1,18 | .74 |
|  | Memory outcome | 0.19 | 1,18 | .67 |
|  | Update method X Classifier evidence | 7.45 | 1,18 | .01 |
|  | Update method X memory outcome | 0.07 | 1,18 | .80 |
|  | Classifier evidence X memory outcome | 18.46 | 1,18 | < .001 |
|  | Update method X Classifier evidence X Memory outcome | 0.89 | 1,18 | .36 |

Supplementary File 2b. Updating method (RetPrac, Restudy) X Memory outcome (Correct, Incorrect) 2-way ANOVA table by Classifier evidence type.

| ROI | Classifier evidence | Effect | F | df | P |
| --- | --- | --- | --- | --- | --- |
| MPFC | Target | Update method | 7.01 | 1,18 | .02 |
|  |  | Memory outcome | 15.10 | 1,18 | .001 |
|  |  | Update method X Memory outcome | 0.04 | 1,18 | .85 |
|  | Competitor | Update method | < .01 | 1,18 | .96 |
|  |  | Memory outcome | 5.09 | 1,18 | .04 |
|  |  | Update method X Memory outcome | 2.53 | 1,18 | .13 |
| VTC | Target | Update method | 3.26 | 1,18 | .09 |
|  |  | Memory outcome | 26.69 | 1,18 | < .001 |
|  |  | Update method X Memory outcome | 2.01 | 1,18 | .17 |
|  | Competitor | Update method | 4.68 | 1,18 | .04 |
|  |  | Memory outcome | 1.71 | 1,18 | .21 |
|  |  | Update method X Memory outcome | 0.37 | 1,18 | .55 |
| AG | Target | Update method | 1.85 | 1,18 | .19 |
|  |  | Memory outcome | 7.23 | 1,18 | .02 |
|  |  | Update method X Memory outcome | .03 | 1,18 | .87 |
|  | Competitor | Update method | 1.87 | 1,18 | .19 |
|  |  | Memory outcome | 6.04 | 1,18 | .02 |
|  |  | Update method X Memory outcome | 0.50 | 1,18 | .49 |
