# Supplementary material for: Retrieval practice facilitates memory updating by enhancing and differentiating medial prefrontal cortex representations
Source: eLife. 2020 May 18;9:e57023. doi: 10.7554/eLife.57023 (PMC7272192; doi:10.7554/eLife.57023)
Supplement: Supplementary file 3. — (a) Regions showing greater activation during updating under the RetPrac condition than under the Restudy condition. (b) Regions showing greater activation during updating under the Restudy condition than under the RetPrac condition. (c) Pairwise comparisons of brain activity between trials with different updating performance. [file elife-57023-supp3.docx]

Supplementary File 3a. Regions showing greater activation during updating under the RetPrac condition than under the Restudy condition.

|  | Coordinates | | |  |
| --- | --- | --- | --- | --- |
| Region | x | y | z | T |
| Left Middle Frontal Gyrus | -38 | 20 | 24 | 7.40 |
| Left Caudate | -8 | 12 | 4 | 12.54 |
| Right Caudate | -2 | 10 | 4 | 13.30 |
| Left Insula Cortex | -32 | 22 | -4 | 12.61 |
| Right Insula Cortex | 34 | 26 | 2 | 13.24 |
| Left Orbital Cortex | -46 | 20 | -8 | 7.45 |
| Right Orbital Cortex | 34 | 26 | 2 | 13.24 |
| Left Frontal Pole | -50 | 42 | 8 | 6.73 |
| Superior Frontal Gyrus | -2 | 16 | 52 | 13.30 |
| Paracingulate Gyrus | 0 | 28 | 44 | 13.71 |
| Left Lateral Occipital Cortex | -32 | -62 | 44 | 8.28 |
| Left Intracalcarine Cortex | -10 | -76 | 10 | 7.07 |
| Right Intracalcarine Cortex | 10 | -72 | 14 | 6.41 |
| Left Precuneous Cortex | -12 | -66 | 32 | 11.94 |

Supplementary File 3b. Regions showing greater activation during updating under the Restudy condition than under the RetPrac condition.

|  | Coordinates | | |  |
| --- | --- | --- | --- | --- |
| Region | x | y | z | T |
| Left Hippocampus | -24 | -18 | -16 | 10.24 |
| Right Hippocampus | 26 | -18 | -16 | 13.35 |
| Left Occipital Fusiform Gyrus | -28 | -70 | -16 | 10.74 |
| Right Occipital Fusiform Gyrus | 34 | -68 | -18 | 12.52 |
| Left Temporal Occipital Fusiform Cortex | -26 | -54 | -16 | 9.20 |
| Right Temporal Occipital Fusiform Cortex | 30 | -54 | -18 | 12.01 |
| Medial Prefrontal Cortex | 2 | 30 | 8 | 10.77 |
|  | 0 | 6 | -8 | 12.43 |
|  | 2 | 38 | -18 | 7.36 |
|  | 2 | 60 | 16 | 9.01 |
| Left Supramarginal Gyrus | -66 | -34 | 22 | 9.85 |
| Right Supramarginal Gyrus | 58 | -30 | 34 | 10.64 |
| Left Angular Gyrus | -62 | -54 | 30 | 9.30 |
| Right Angular Gyrus | 60 | -56 | 18 | 9.39 |
| Left Middle Temporal Gyrus | -66 | -14 | -8 | 9.98 |
| Right Middle Temporal Gyrus | 54 | -4 | -32 | 9.34 |
| Left Temporal Pole | -50 | 16 | -32 | 7.41 |
| Right Temporal Pole | 54 | 4 | -38 | 10.54 |
| Left Lateral Occipital Cortex, inferior division | -52 | -70 | -4 | 9.60 |
| Right Lateral Occipital Cortex, inferior division | 52 | -68 | -4 | 8.76 |
| Left Lateral Occipital Cortex, superior division | -42 | -78 | 16 | 6.74 |
| Right Lateral Occipital Cortex, superior division | 34 | -88 | 12 | 9.83 |
| Left Insular Cortex | -40 | -14 | -6 | 12.03 |
| Right Insular Cortex | 42 | -8 | -10 | 11.82 |
| Cingulate Gyrus, posterior division | 2 | -22 | 46 | 10.69 |

Supplementary File 3c. Pairwise comparisons of brain activity between trials with different updating performance.

|  | LC vs. FC | | LC vs. IC | | FC vs. IC | |
| --- | --- | --- | --- | --- | --- | --- |
| Region | T | P | T | P | T | P |
| Left DLPFC | -7.17 | < .001 | -4.76 | < .001 | 0.66 | .52 |
| dACC | -6.44 | < .001 | -4.89 | < .001 | 0.21 | .84 |
| Left anterior insular | -5.88 | < .001 | -5.19 | < .001 | 0.53 | .60 |
| Right anterior insular | -5.53 | < .001 | -3,71 | < .001 | 0.53 | .60 |
| Left caudate | -4.73 | < .001 | 1.28 | .22 | 4.82 | < .001 |
| Right caudate | -3.34 | .004 | 1.81 | .09 | 5.03 | < .001 |

Note: FC, first correct trials; LC: later correct trials; IC: incorrect trials
